# Activation of cancerous inhibitor of PP2A (CIP2A) contributes to lapatinib resistance through induction of CIP2A-Akt feedback loop in ErbB2-positive breast cancer cells

**DOI:** 10.18632/oncotarget.19375

**Published:** 2017-07-19

**Authors:** Ming Zhao, Erin W. Howard, Amanda B. Parris, Zhiying Guo, Qingxia Zhao, Zhikun Ma, Ying Xing, Bolin Liu, Susan M. Edgerton, Ann D. Thor, Xiaohe Yang

**Affiliations:** ^1^ Julius L. Chambers Biomedical/Biotechnology Research Institute and Department of Biological and Biomedical Sciences, North Carolina Central University, Kannapolis, North Carolina, USA; ^2^ Basic Medical College of Zhengzhou University, Zhengzhou, Henan, P.R. China; ^3^ Department of Pathology, School of Medicine, University of Colorado Anschutz Medical Campus, Aurora, Colorado, USA; ^4^ College of Medicine, Henan University of Sciences and Technology, Luoyang, Henan, P.R. China

**Keywords:** CIP2A, lapatinib resistance, ErbB2, breast cancer, PP2A

## Abstract

Lapatinib, a small molecule ErbB2/EGFR inhibitor, is FDA-approved for the treatment of metastatic ErbB2-overexpressing breast cancer; however, lapatinib resistance is an emerging clinical challenge. Understanding the molecular mechanisms of lapatinib-mediated anti-cancer activities and identifying relevant resistance factors are of pivotal significance. Cancerous inhibitor of protein phosphatase 2A (CIP2A) is a recently identified oncoprotein that is overexpressed in breast cancer. Our study investigated the role of CIP2A in the anti-cancer efficacy of lapatinib in ErbB2-overexpressing breast cancer cells. We found that lapatinib concurrently downregulated CIP2A and receptor tyrosine kinase signaling in ErbB2-overexpressing SKBR3 and 78617 cells; however, these effects were attenuated in lapatinib-resistant (LR) cells. CIP2A overexpression rendered SKBR3 and 78617 cells resistant to lapatinib-induced apoptosis and growth inhibition. Conversely, CIP2A knockdown via lentiviral shRNA enhanced cell sensitivity to lapatinib-induced growth inhibition and apoptosis. Results also suggested that lapatinib downregulated CIP2A through regulation of protein stability. We further demonstrated that lapatinib-induced CIP2A downregulation can be recapitulated by LY294002, suggesting that Akt mediates CIP2A upregulation. Importantly, lapatinib induced differential CIP2A downregulation between parental BT474 and BT474/LR cell lines. Moreover, CIP2A shRNA knockdown significantly sensitized the BT474/LR cells to lapatinib. Collectively, our results demonstrate that CIP2A is a molecular target and resistance factor of lapatinib with a critical role in lapatinib-induced cellular responses, including the inhibition of the CIP2A-Akt feedback loop. Further investigation of lapatinib-mediated CIP2A regulation will advance our understanding of lapatinib-associated anti-tumor activities and drug resistance.

## INTRODUCTION

ErbB2 is a receptor tyrosine kinase (RTK) belonging to the epidermal growth factor receptor (EGFR) family, which is comprised of four members: EGFR/ErbB1, ErbB2/Her2/Neu, ErbB3, and ErbB4 [1, 2]. Amplification of the *ERBB2* oncogene is detected in approximately 25–30% of invasive breast cancers, which has been associated with a more aggressive phenotype, poor prognosis, and chemoresistance [3]. ErbB2-mediated carcinogenesis has been attributed to the activation of a plethora of downstream pathways involved in cell proliferation, survival, and angiogenesis, such as the PI3K/Akt and MAPK/Erk pathways [4–6]. ErbB2 is the only EGFR family member that has no known binding ligand; hence, the activation of ErbB2 depends largely on heterodimerization with other family members upon the binding of their cognate ligands. This interaction induces autophosphorylation of specific tyrosine residues within the catalytic kinase domain and triggers downstream cell signaling pathways [7]. Extensive studies have established ErbB2 as a valid therapeutic target. As such, clinical implementation of therapeutic agents targeting ErbB2, including trastuzumab and lapatinib, has achieved remarkable benefits in patients with ErbB2-overexpressing breast cancer; however, the development of resistance to these novel agents is emerging as a significant clinical challenge.

Lapatinib is a small molecule dual inhibitor targeting both ErbB2 and EGFR. It reversibly binds to the cytoplasmic ATP-binding site of the kinases and blocks receptor phosphorylation and activation, thereby preventing subsequent downstream signaling events [8]. Preclinical studies have shown that lapatinib inhibits cell proliferation in EGFR and/or ErbB2-overexpressing breast cancer cell lines, even in trastuzumab-resistant cells [9]. Likewise, the combination of lapatinib and trastuzumab synergistically inhibits ErbB2-overexpressing cell lines [10]. Lapatinib is FDA-approved to treat ErbB2-positive (ErbB2^+^) advanced or metastatic breast cancer, and its use, either alone or in combination with trastuzumab, capecitabine, or other agents, has achieved significant improvement in clinical outcomes [11, 12]. Nevertheless, the development of lapatinib resistance hinders the efficacy of this promising drug. Hence, understanding the mechanisms of lapatinib-induced tumor inhibition and identifying the factors that contribute to lapatinib resistance is of pivotal significance in clinical oncology.

Previous studies have shown that lapatinib resistance can be induced by different mechanisms, including the activation of various RTKs and intracellular kinases [13]. For example, Garrett and colleagues (2011) demonstrated that the induction of FoxO3A-dependent upregulation of ErbB3/Her3 causes lapatinib resistance [14]. Activation of HGF/MET signaling also contributes to sustained resistance to ErbB2-targeting therapies [15]. Moreover, alterations in intracellular kinases, such as Src and mTOR, allow the cells to circumvent ErbB2 blockage [16, 17]. Lapatinib resistance has also been attributed to the overexpression of ErbB ligands, such as neuregulin-1 and heregulin, and crosstalk between ErbB2 and estrogen receptor (ER) pathways [18–20]. More recently, Stuhlmiller *et al*. (2015) demonstrated that lapatinib elicits highly heterogeneous and adaptive kinome reprogramming, involving ErbB3, IGF1R, DDR1, MET, and FGFRs, which consequently led to lapatinib resistance. Further, they reported that the combination of kinase inhibitors and chromatin reader inhibitors prevents kinome adaptation and more effectively overcomes lapatinib resistance [21]. Taken together, these reports indicate that acquired lapatinib resistance involves various compensatory pathways that elude lapatinib-mediated cellular responses. Identification of novel factors that provide compensatory signaling will lead to the development of drugs or therapeutic regimens that overcome lapatinib resistance.

Cancerous inhibitor of protein phosphatase 2A (CIP2A) is a newly identified oncogenic protein that acts as an endogenous inhibitor of PP2A. CIP2A can inhibit PP2A-mediated c-Myc dephosphorylation at Ser62 and thereby stabilize the c-Myc protein [22]. Through the abrogation of PP2A activity, CIP2A is further involved in the regulation of other signaling proteins/pathways, including Akt, p53, MEK1, E2F1, DAPk, and PLK1 [23, 24]. Based on the broad-reaching interactions between CIP2A and these critical regulators, De *et al*. (2014) proposed the ‘oncogenic nexus’ concept, which highlights the critical role of CIP2A in the regulation of cell proliferation, survival, and malignant transformation [23]. Data based on clinical samples reveal that CIP2A is overexpressed in various types of cancers, including breast cancer, and is indicative of poor prognostic outcomes [22, 25, 26]. As such, Côme *et al*. (2009) found that CIP2A is associated with clinical aggressiveness and promotes malignant growth in breast cancer patients [26]. Moreover, CIP2A regulation is involved in responses to chemotherapy and targeted therapeutic agents, such as doxorubicin, erlotinib, and bortezomib [27–29]. A recent report indicated that lapatinib mediates CIP2A inhibition, Akt inactivation, and apoptosis induction in HCC 1937, MDA-MB-468, and MDA-MB-231 triple-negative breast cancer cells [30]. Together, these data suggest that CIP2A may serve as a promising target in molecular therapeutics due to its important role in breast cancer etiology. Given its interactions with the PP2A network and oncogenic signaling pathways, CIP2A regulation in drug efficacy and acquired therapeutic resistance warrants further investigation.

In this study, we investigated the effect of CIP2A on lapatinib-induced anti-cancer activities and resistance in ErbB2-overexpressing breast cancer cells. We found that CIP2A downregulation correlated with lapatinib-induced apoptosis. Moreover, lapatinib-induced CIP2A downregulation involves Akt-dependent CIP2A degradation through the proteasomal pathway. Importantly, lapatinib stimulated differential CIP2A downregulation between parental and lapatinib-resistant (LR) BT474 cell lines. Indeed, CIP2A knockdown significantly sensitized BT474/LR cells to lapatinib-induced growth inhibition and apoptosis. Our results demonstrate that CIP2A, as an intracellular molecular target of lapatinib, inhibits lapatinib-induced cellular responses and confers therapeutic resistance.

## RESULTS

### Lapatinib-induced CIP2A downregulation is correlated with growth inhibition, apoptosis induction, and RTK signaling inactivation

**Figure 1:**
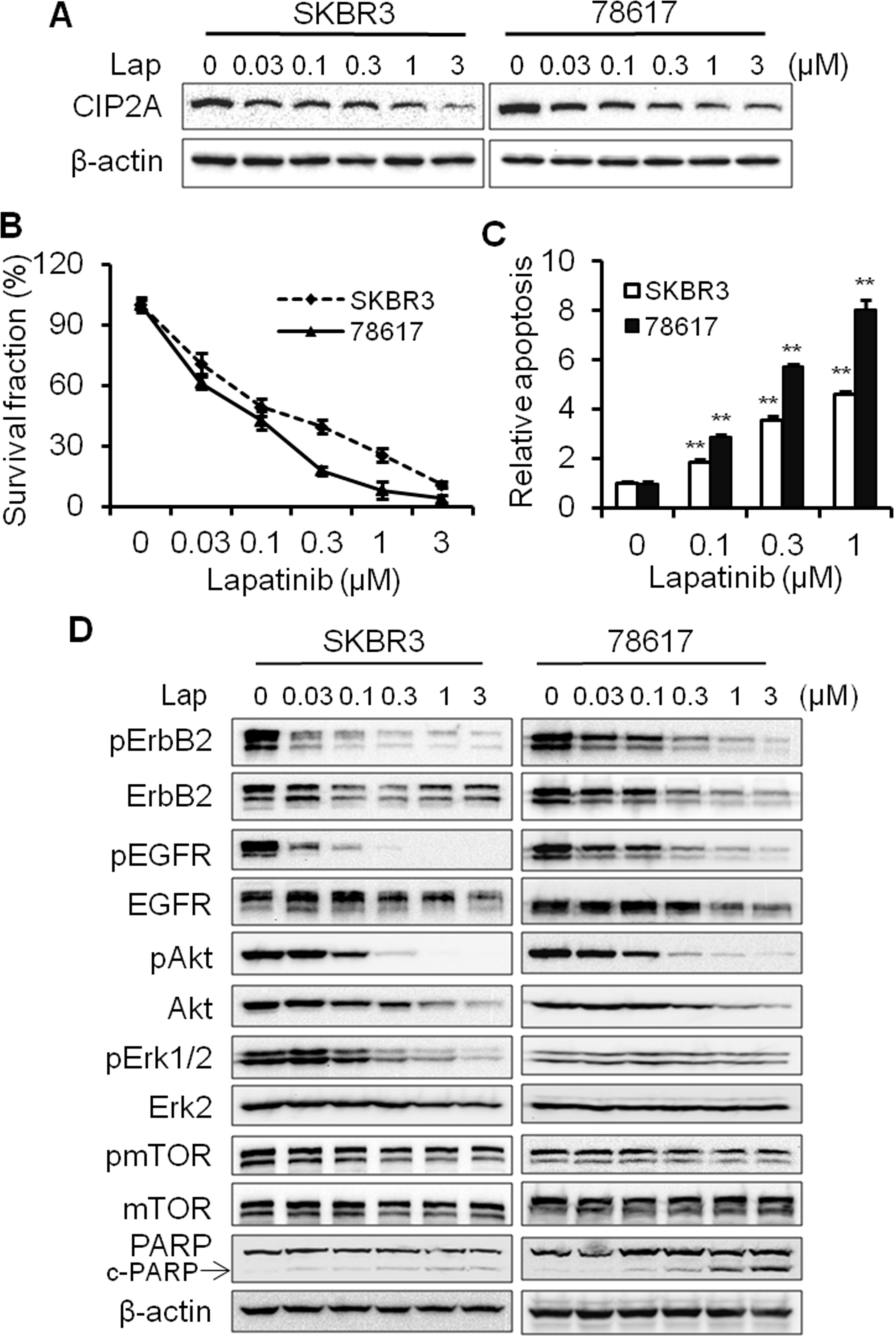
Lapatinib-induced CIP2A downregulation is correlated with growth inhibition, apoptosis induction, and RTK signaling inactivation (**A**) SKBR3 and 78617 cells were treated with lapatinib for 16 hours and CIP2A levels were detected by Western blotting. (**B**) SKBR3 and 78617 cells were treated with indicated concentrations of lapatinib for 5 days, followed by an MTS assay. Results are expressed as the relative viability compared to control cells. (**C**) SKBR3 and 78617 cells were treated with lapatinib for 24 hours, followed by apoptosis detection with a cell death ELISA kit. Relative apoptosis was expressed as a ratio compared to the control. All experiments were performed in triplicate. (**D**) SKBR3 and 78617 cells were treated with lapatinib as in panel A, then the expression of phospho-ErbB2 (Tyr1221/1222), ErbB2, phospho-EGFR (Tyr1068), EGFR, phospho-Akt (Ser473), Akt, phospho-Erk1/2 (Thr202/Tyr204), Erk2, phospho-mTOR (Ser2448), and mTOR were analyzed using Western blotting. All values are presented as the mean ± standard error (S.E.) (**p < 0.01).

It was shown that CIP2A modulation was associated with cellular responses to certain therapeutic agents [26]. Therefore, we examined the role of CIP2A in lapatinib-induced anti-cancer effects in ErbB2-overexpressing SKBR3 human breast cancer cells and 78617 cells, which are derived from spontaneous mammary tumors of MMTV-ErbB2 transgenic mice [31]. Lapatinib downregulated CIP2A protein expression in a dose-dependent manner in both cell lines (Figure 1A). This change was also correlated with cell growth inhibition and apoptosis induction (Figure 1B–1C). In the context of the above findings, key markers of ErbB2/EGFR-mediated signaling pathway were detected. As shown in Figure 1D, lapatinib treatment induced the concurrent downregulation of ErbB2, EGFR, Akt, Erk, and mTOR phosphorylation. To note, lapatinib also dose-dependently suppressed the expression of unphosphorylated ErbB2, EGFR, and Akt. These findings may be the result of lapatinib-induced Hsp90 inhibition, which has previously been reported [32, 33]. Alternatively, lapatinib may induce transcriptional regulation of these markers via modulation of signaling downstream of EGFR and ErbB2. Nevertheless, our protein analyses in the ErbB2-overexpressing breast cancer cells indicate a close correlation between CIP2A downregulation and RTK signaling inhibition.

### CIP2A overexpression renders SKBR3 and 78617 breast cancer cells resistant to lapatinib

**Figure 2:**
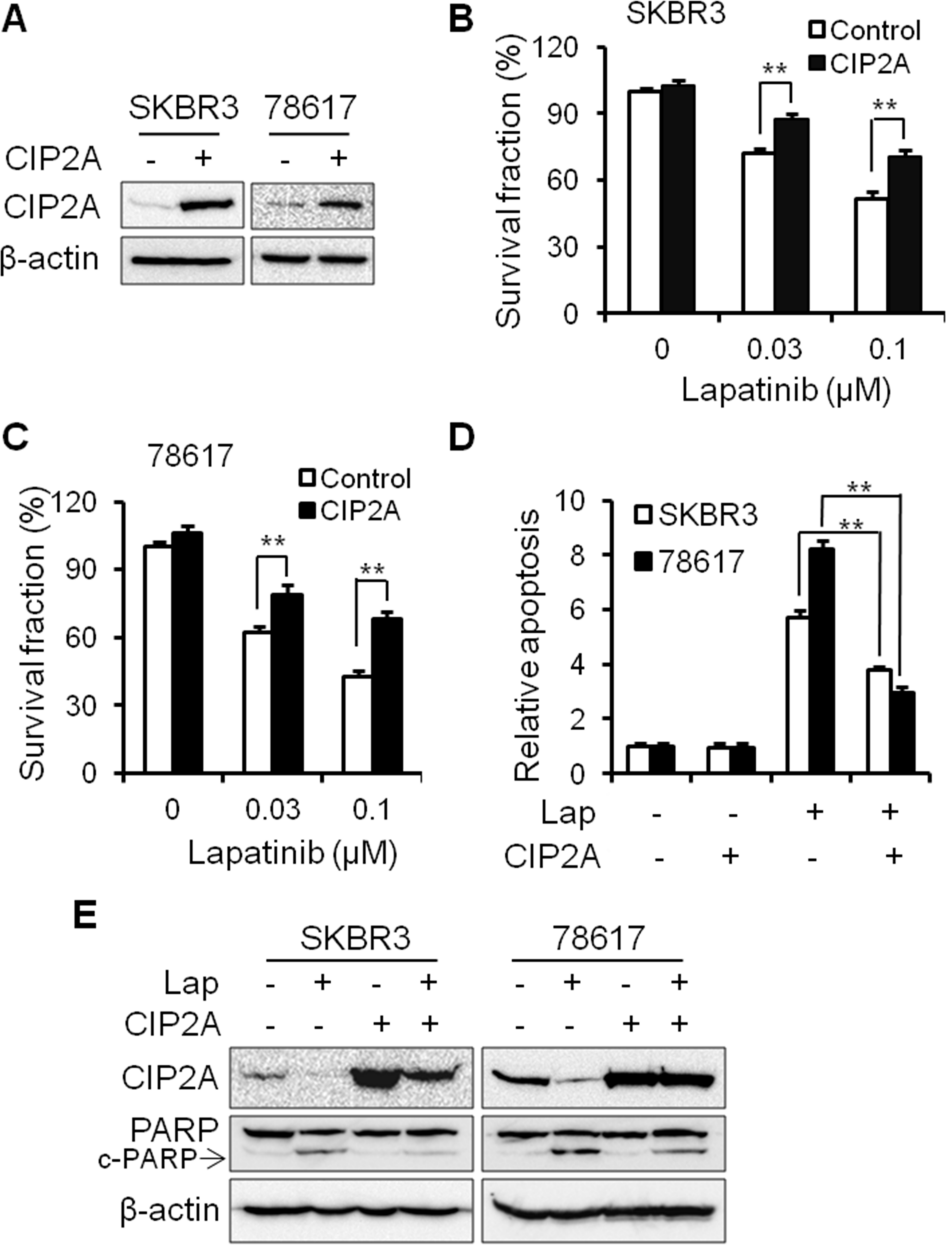
CIP2A overexpression renders SKBR3 and 78617 breast cancer cells resistant to lapatinib (**A**) Relative CIP2A levels in stable SKBR3 and 78617 sublines transfected with control (–) or CIP2A-encoding (+) lentiviral vectors were detected with Western blotting. Survival fractions in control and CIP2A-overexpressing SKBR3 (**B**) and 78617 (**C**) cells treated with lapatinib for 5 days were measured with an MTS assay. Control and CIP2A-overexpressing SKBR3 and 78617 cells were treated with 0.3 μM lapatinib for 24 hours, followed by an apoptosis ELISA assay (**D**) and Western blot analysis of PARP and cleaved PARP (c-PARP) protein expression (**E**). All values are presented as the mean ± S.E. (**p < 0.01).

In order to investigate the functional role of CIP2A in lapatinib-induced cellular responses, we examined the effects of CIP2A overexpression on lapatinib-induced growth inhibition and apoptosis in SKBR3 and 78617 cells. As shown in Figure 2A, the transfection of SKBR3 and 78617 cells with CIP2A-encoding lentivirus resulted in CIP2A overexpression in both cell lines. Data from an MTS assay indicated that CIP2A overexpression attenuates lapatinib-induced growth inhibition (Figure 2B–2C). To determine the effect of CIP2A overexpression on lapatinib-induced apoptosis, we assessed lapatinib-treated control and CIP2A-overexpressing cells with a cell death ELISA assay. We found that lapatinib-induced apoptosis in CIP2A-overexpressing cells was significantly reduced as compared to the lapatinib-treated cells expressing endogenous CIP2A levels (Figure 2D). These results were supported by the decrease of PARP cleavage in lapatinib-treated CIP2A-overexpressing SKBR3 and 78617 cells (Figure 2E). Our data indicate that CIP2A overexpression is associated with resistance to lapatinib-induced growth inhibition and apoptosis induction.

### CIP2A knockdown enhances SKBR3 and 78617 cell sensitivity to lapatinib

**Figure 3:**
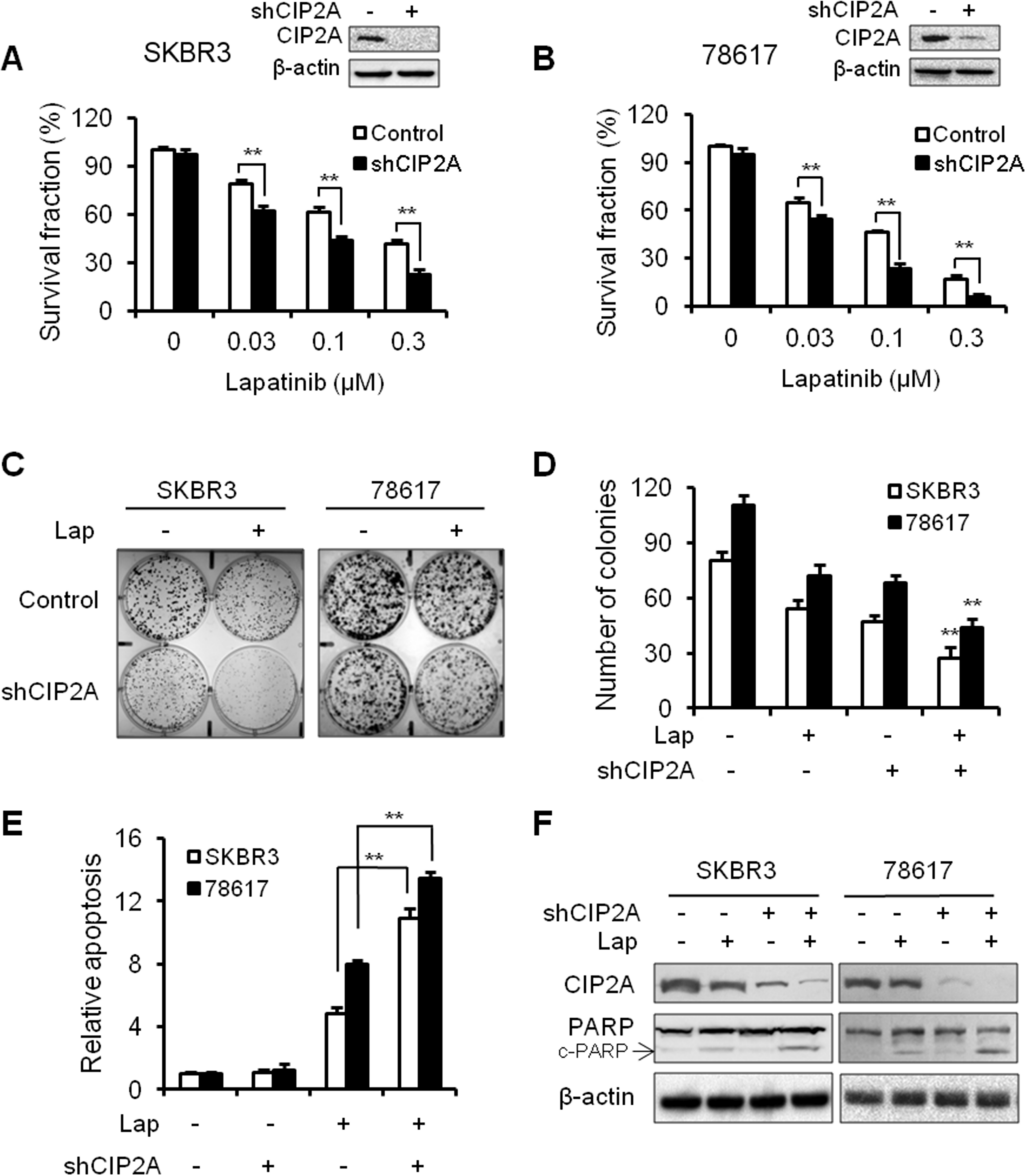
CIP2A knockdown enhances SKBR3 and 78617 cell sensitivity to lapatinib Survival fractions in control and CIP2A knockdown SKBR3 (**A**) and 78617 (**B**) cells treated with lapatinib for 5 days were measured with an MTS assay. Inserts show the knockdown efficiency of transient transfection. Control and CIP2A knockdown SKBR3 and 78617 cells were treated with 0.03 μM lapatinib for 2 weeks, then clonogenic survival was determined by staining colonies with crystal violet. Representative images are shown in (**C**). The bar graph represents the mean values from three independent experiments (**D**). Lapatinib-induced (0.3 μM for 24 hours) apoptosis in control and CIP2A knockdown cells was detected with a cell death ELISA assay (**E**) and Western blot analysis of PARP cleavage (**F**). All values are presented as the mean ± S.E. (**p < 0.01).

To confirm the role of CIP2A in cellular responses to lapatinib, we examined the effect of CIP2A shRNA knockdown on lapatinib-induced growth inhibition and apoptosis. As shown in Figure 3A–3B, treatment of SKBR3 and 78617 cells with CIP2A shRNA-encoding lentiviral vectors resulted in the effective knockdown of CIP2A protein expression, which significantly enhanced lapatinib-induced growth inhibition in both cell lines. Consistently, lapatinib-induced growth inhibition was also confirmed by a clonogenic assay. CIP2A knockdown alone resulted in a moderate decrease in the colony forming capacity of both cell lines, while the colony numbers were significantly decreased in CIP2A knockdown cells treated with lapatinib as compared to the cells expressing endogenous CIP2A (Figure 3C–3D). Furthermore, results based on a cell death ELISA assay and PARP cleavage demonstrated that lapatinib-induced apoptosis in the cells with CIP2A knockdown was significantly increased when compared with corresponding controls (Figure 3E–3F). Taken together, these data demonstrate that endogenous CIP2A can interfere with lapatinib-induced growth inhibition and apoptosis.

### Lapatinib induces CIP2A degradation in an Akt-dependent manner through the proteasomal pathway

**Figure 4:**
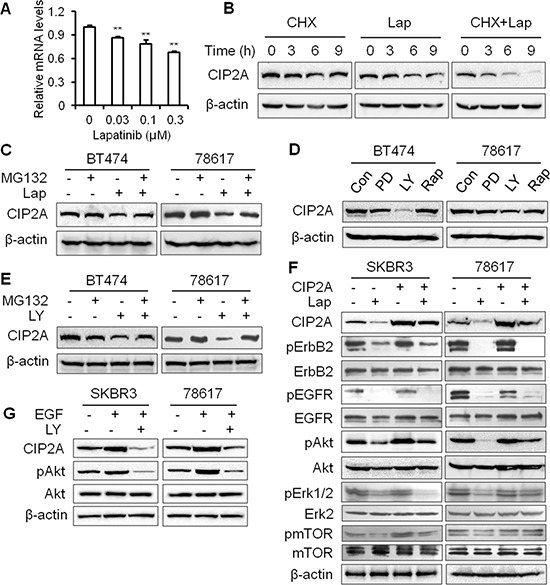
Lapatinib induces CIP2A degradation in an Akt-dependent manner through the proteasomal pathway (**A**) *CIP2A* mRNA levels in BT474 cells after lapatinib treatment for 16 hours were measured by qPCR. All values are presented as the mean ± S.E (**p < 0.01 as compared to the control). (**B**) BT474 cells were pretreated with CHX (20 μg/mL), lapatinib (0.3 μM) alone, or in combination, followed by Western blot analysis of CIP2A protein levels after 0 – 9 hours of indicated treatments. (**C**) BT474 and 78617 cells were pretreated with MG132 (2 μM) alone for 1 hour, lapatinib (0.3 μM) alone for 12 hours, or in combination, then CIP2A levels were measured by Western blotting. (**D**) BT474 and 78617 cells were treated with vehicle control (DMSO), PD98059 (20 μM; Erk inhibitor), LY294002 (20 μM; PI3K/Akt inhibitor), or rapamycin (50 nM; mTOR inhibitor) for 16 hours, followed by CIP2A detection with Western blotting. (**E**) BT474 and 78617 cells were pretreated with MG132 (2 μM) alone for 1 hour, LY294002 (20 μM) alone for 12 hours, or in combination before Western blot analysis of CIP2A. (**F**) SKBR3 and 78617 cells were transfected with control or CIP2A-encoding lentiviral vectors and then treated with lapatinib (0.3 μM) for 16 hours. The indicated proteins were assessed by Western blotting. (**G**) SKBR3 and 78617 cells were serum starved for 24 hours and then were stimulated with EGF (50 ng/mL) alone for 1 hour or in combination with LY294002 (20 μM) for 1 hour before Western blot analysis.

To investigate the mechanisms of lapatinib-induced CIP2A downregulation, we examined the mRNA and protein levels of CIP2A in lapatinib-treated cells. As shown in Figure 4A, lapatinib treatment induced a significant decrease in *CIP2A* mRNA levels in BT474 cells, which is consistent with previous reports indicating that lapatinib inhibited the binding of transcription factors to the CIP2A promoter [30]. In the presence of cycloheximide (CHX), which blocks protein synthesis, CIP2A degradation was more evident in lapatinib-treated BT474 cells as compared to the control cells (Figure 4B, Supplementary Figure 1A). These results indicate that lapatinib downregulated CIP2A at the mRNA and protein levels. We then focused on the mechanisms of lapatinib-induced CIP2A degradation. To confirm lapatinib-induced CIP2A protein degradation through the proteasomal pathway, we further demonstrated that MG132, a potent protease inhibitor, attenuated lapatinib-induced CIP2A downregulation in BT474 and 78617 cells (Figure 4C, Supplementary Figure 1B). MG132 also suppressed lapatinib-mediated CIP2A degradation, as indicated by the accumulation of ubiquitinated CIP2A in lapatinib-treated cells (Supplementary Figure 2). Since MAPK/Erk, PI3K/Akt, and mTOR signaling inhibition is a major lapatinib-induced cellular response, we used PD98059, LY294002, and rapamycin to test the specific role of the Erk, Akt, and mTOR pathways in CIP2A modulation, respectively. Among the three inhibitors, only the PI3K specific inhibitor, LY294002, resulted in a significant decrease in CIP2A protein expression (Figure 4D, Supplementary Figure 1C). Moreover, MG132 treatment was able to block LY294002-induced CIP2A downregulation (Figure 4E, Supplementary Figure 1D). Together, these results indicate that the suppression of Akt kinase activity induces CIP2A degradation through the proteasomal pathway, which may contribute to lapatinib-induced CIP2A degradation. Consistent with a previous report that CIP2A promotes Akt activation through the inhibition of PP2A-mediated regulation of Akt kinase activity [29], we demonstrated that CIP2A overexpression significantly increased phosphorylation levels of Akt and mTOR in SKBR3 cells and to a lesser extent in 78617 cells (Figure 4F, Supplementary Figure 1E). Lapatinib was also capable of reducing CIP2A overexpression-induced activation of Akt and mTOR in SKBR3 and 78617 cells. Moreover, LY294002 inhibited the enhanced levels of CIP2A and phosphorylated Akt induced by EGF, which suggests a regulatory link between CIP2A and Akt (Figure 4G, Supplementary Figure 1F). On the basis that ErbB2 can heterodimerize with other EGFR family members [6], EGFR/ErbB2 activation appears to be inherently involved in this CIP2A-Akt feedback mechanism. Taken together, we demonstrated the presence of a positive feedback loop between CIP2A and Akt. In turn, lapatinib-induced inhibition of EGFR and ErbB2 suppresses this feedback loop and downregulates Akt and CIP2A simultaneously.

### CIP2A-c-Myc pathway is involved in lapatinib-induced cell cycle regulation

**Figure 5:**
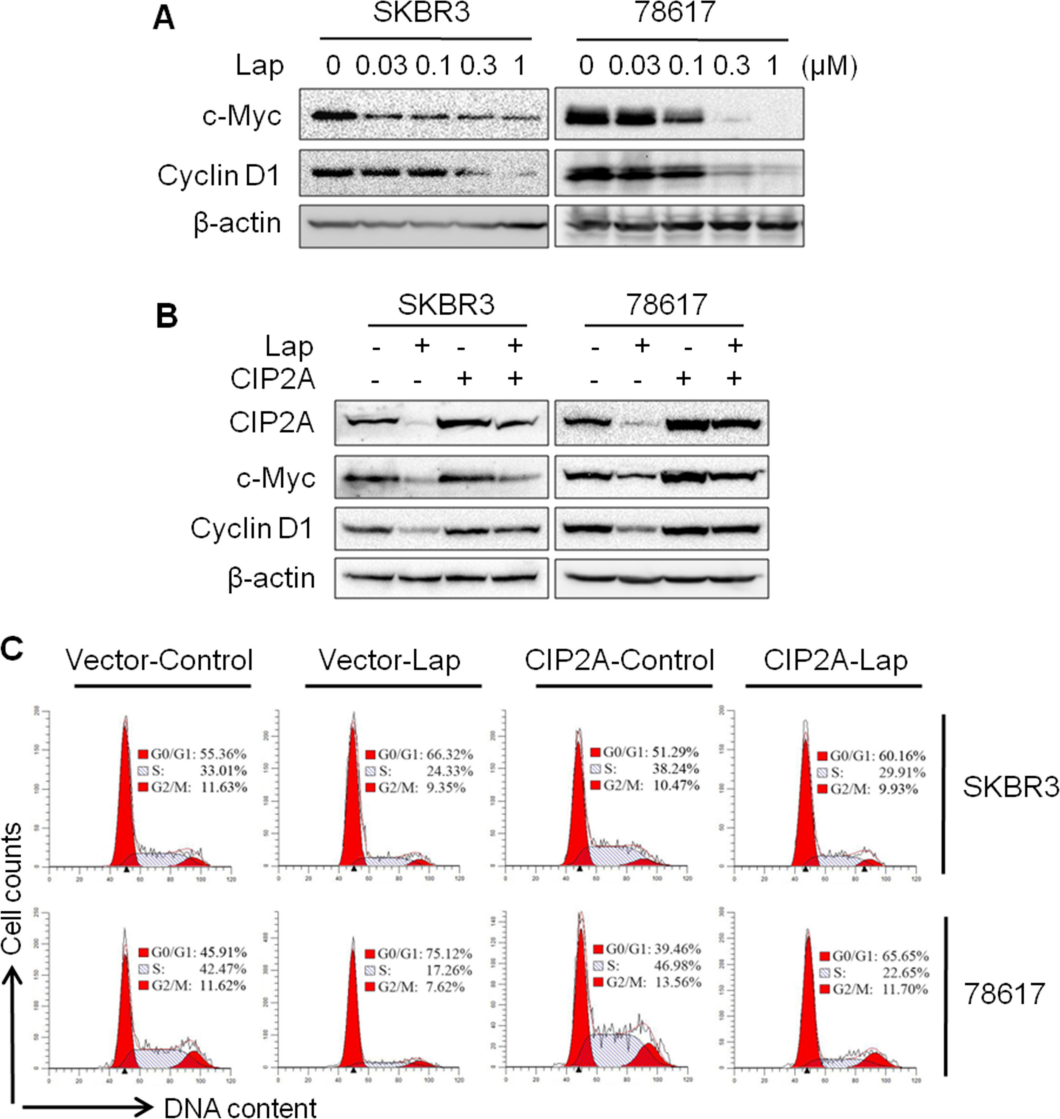
CIP2A/c-Myc pathway is involved in lapatinib-induced cell cycle regulation (**A**) SKBR3 and 78617 cells were treated with lapatinib for 16 hours, followed by Western blot analysis. Control or CIP2A-overexpressing SKBR3 and 78617 cells were treated with lapatinib (0.3 μM) for 16 hours, then examined using Western blot (**B**) and cell cycle (**C**) analyses.

Previous studies have shown that CIP2A stabilizes c-Myc through the inhibition of PP2A-mediated dephosphorylation of c-Myc at Ser62, which is required for CIP2A-associated oncogenic activities [22]. As c-Myc and its target genes regulate cell cycle progression, apoptosis, metabolism, and cell adhesion, we examined the role of CIP2A in lapatinib-induced regulation of the c-Myc pathway. As shown in Figure 5A, lapatinib treatment resulted in a remarkable dose-dependent decrease in c-Myc and cyclin D1, a classic target of c-Myc. Importantly, we demonstrated that CIP2A overexpression not only increased c-Myc and cyclin D1 expression, but also mitigated lapatinib-induced downregulation of c-Myc and cyclin D1 (Figure 5B, Supplementary Figure 3). The results of cell cycle analysis revealed that CIP2A overexpression increased the percentage of cells in S phase and attenuated the G0/G1 cell cycle arrest stimulated by lapatinib (Figure 5C). Alongside the growth inhibition data depicted in Figure 1B, these results suggest that the suppression of c-Myc and cyclin D1 may be an important factor in lapatinib-induced growth inhibition, and CIP2A downregulation may act as a mediator of this regulation.

### CIP2A overexpression confers lapatinib resistance in breast cancer cells

**Figure 6:**
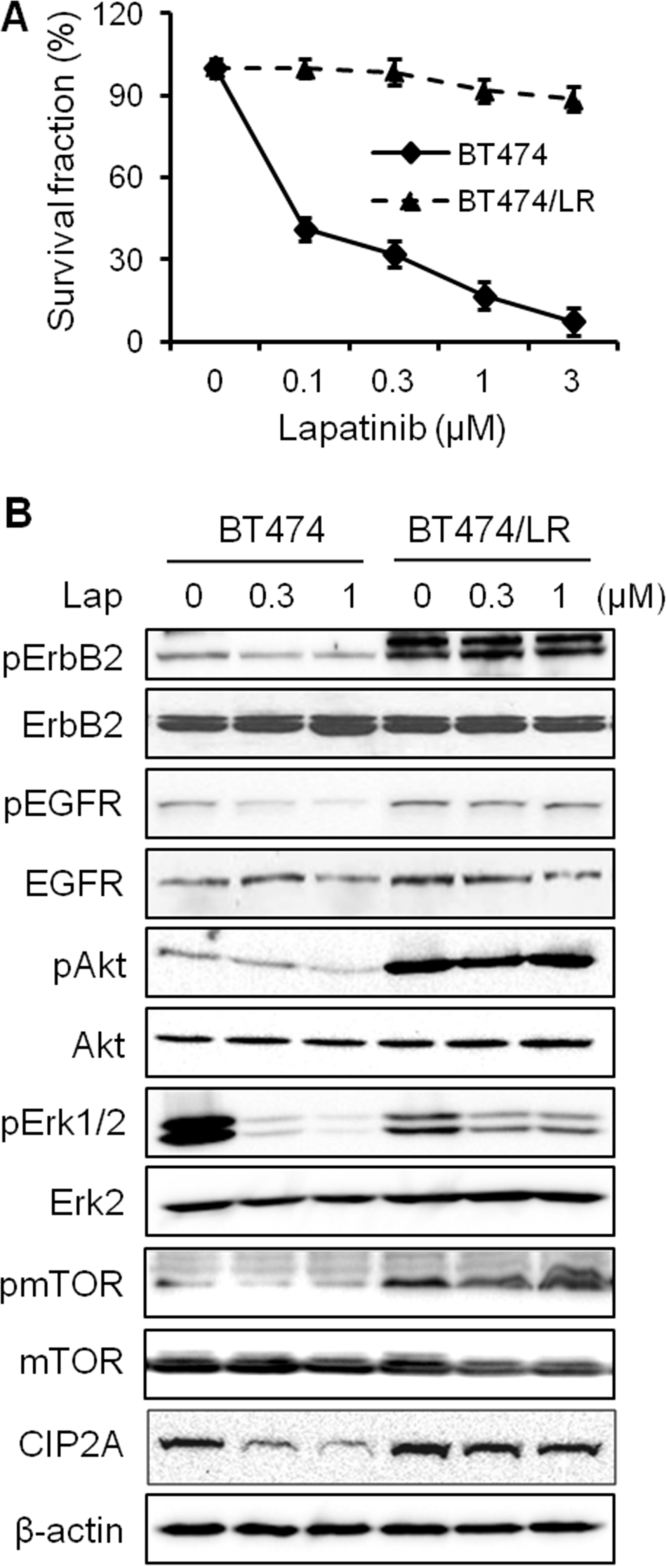
CIP2A overexpression confers lapatinib resistance in breast cancer cells (**A**) Survival fraction of BT474 and BT474/LR cells after lapatinib treatment for 5 days was examined by an MTS assay. All values are presented as the mean ± S.E. (**B**) RTK and CIP2A expression and/or activity levels in BT474 and BT474/LR cells treated with lapatinib for 24 hours were detected by Western blotting.

**Figure 7:**
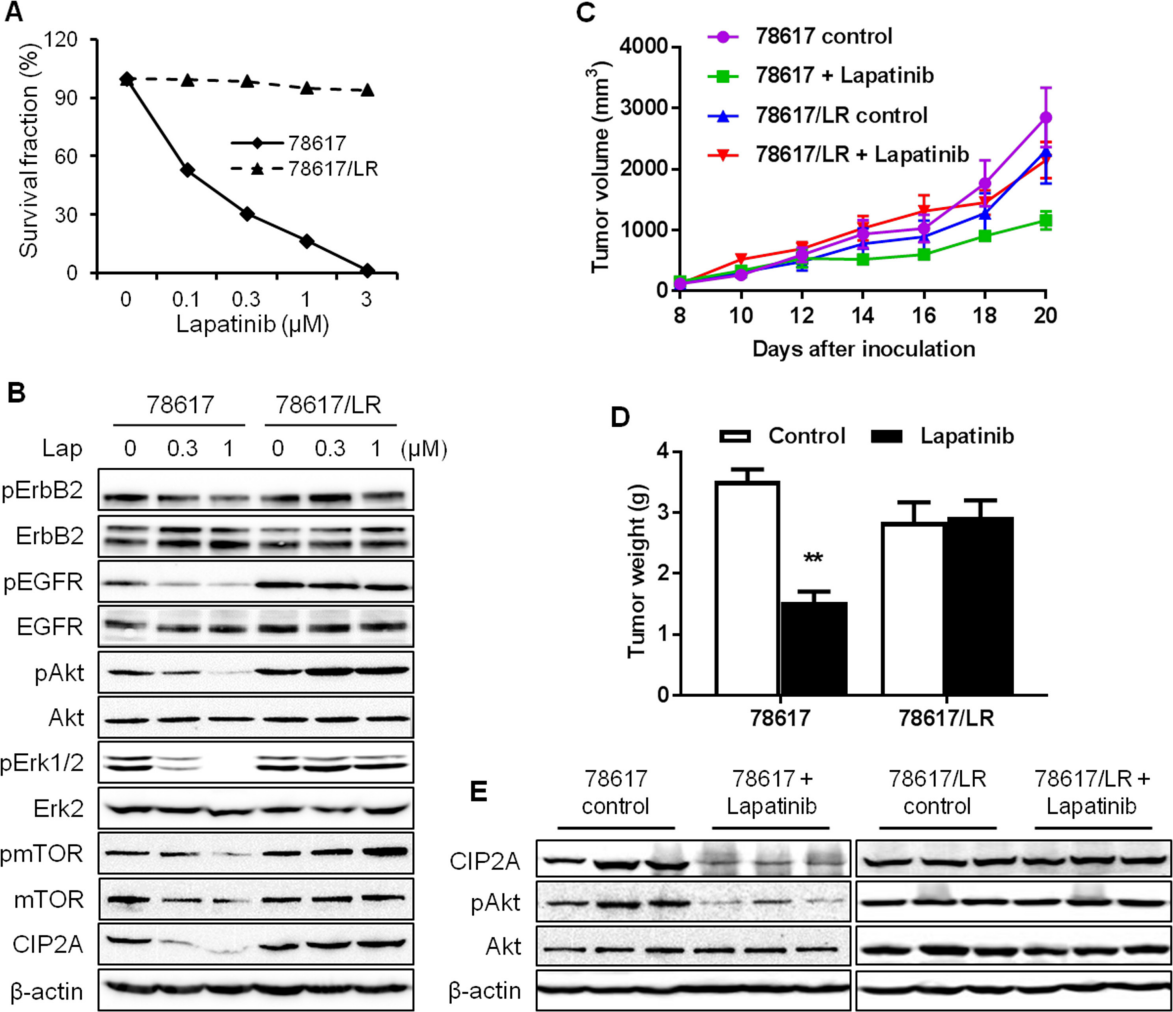
*In vitro* and *in vivo* characterization of lapatinib resistance in 78617/LR cells (**A**) Survival fraction of 78617 and 78617/LR cells after lapatinib treatment for 5 days was examined by an MTS assay. (**B**) RTK and CIP2A expression and/or activity levels in 78617 and 78617/LR cells treated with lapatinib for 24 hours were detected by Western blotting. 78617 and 78617/LR cells were inoculated in the flanks of MMTV-ErbB2 transgenic mice for syngeneic tumor transplantation experiments. Approximately 6 days after tumor cell inoculations, palpable tumors were formed and lapatinib (75 mg/kg BW twice daily via oral gavage) treatments began. Tumor growth was monitored every 2 days and tumor dimensions were recorded. Tumor volumes throughout the duration of lapatinib treatments are graphed in (**C**). After 12 days of lapatinib treatment, tumors were excised and weighed. The average tumor weights are graphed in (**D**). (**E**) Total protein was extracted from collected tumors after 4 days of lapatinib treatments and Western blotting was performed to detect CIP2A levels and Akt activation and expression. All values are presented as the mean ± S.E. (**p < 0.01).

Data from above experiments demonstrate that CIP2A downregulation enhances lapatinib-induced effects on signal transduction and cellular responses. Clinical data further indicate that CIP2A overexpression is frequently detected in breast and other types of cancers, which are associated with poor clinical outcomes [22, 25, 26]. We therefore investigated a possible connection between CIP2A deregulation and lapatinib resistance. To this end, we developed a lapatinib-resistant BT474 (BT474/LR) subline by chronic exposure of parental BT474 cells to increasing concentrations of lapatinib over 5 months and the derived subline was resistant to lapatinib concentrations up to 4 μM. As shown in Figure 6A, lapatinib treatment of the BT474 parental and BT474/LR cells resulted in striking differences in survival fractions. The lapatinib-resistant phenotype in the BT474/LR cells was further confirmed based on the diminished lapatinib-induced inhibition of ErbB2, EGFR, Akt, Erk, and mTOR activation (Figure 6B). Importantly, CIP2A protein levels in lapatinib-treated cells showed different patterns between the two cell lines. In contrast to significant CIP2A downregulation in lapatinib-treated parental cells, the effect of lapatinib on CIP2A expression in BT474/LR cells was also minimal, suggesting a correlation between lapatinib resistance and aberrant CIP2A regulation. We also validated the lapatinib-resistant phenotype using 78617 cells and a 78617/LR subline. Indeed, the 78617/LR cells demonstrated similar resistance as compared to the BT474/LR cells as shown in MTS and Western blot analyses (Figure 7A–7B). *In vivo* data using syngeneic tumor cell transplantation indicated that tumor growth and final tumor weight in the 78617/LR-derived tumors were not significantly affected by lapatinib treatment (75 mg/kg BW twice daily) (Figure 7C–7D). Additionally, lapatinib suppressed CIP2A expression and Akt activation in the 78617 tumors, but not in the 78617/LR tumors (Figure 7E). Together, these data provide *in vitro* and *in vivo* evidence of the effects of lapatinib resistance on cell/tumor growth and RTK signal transduction.

**Figure 8:**
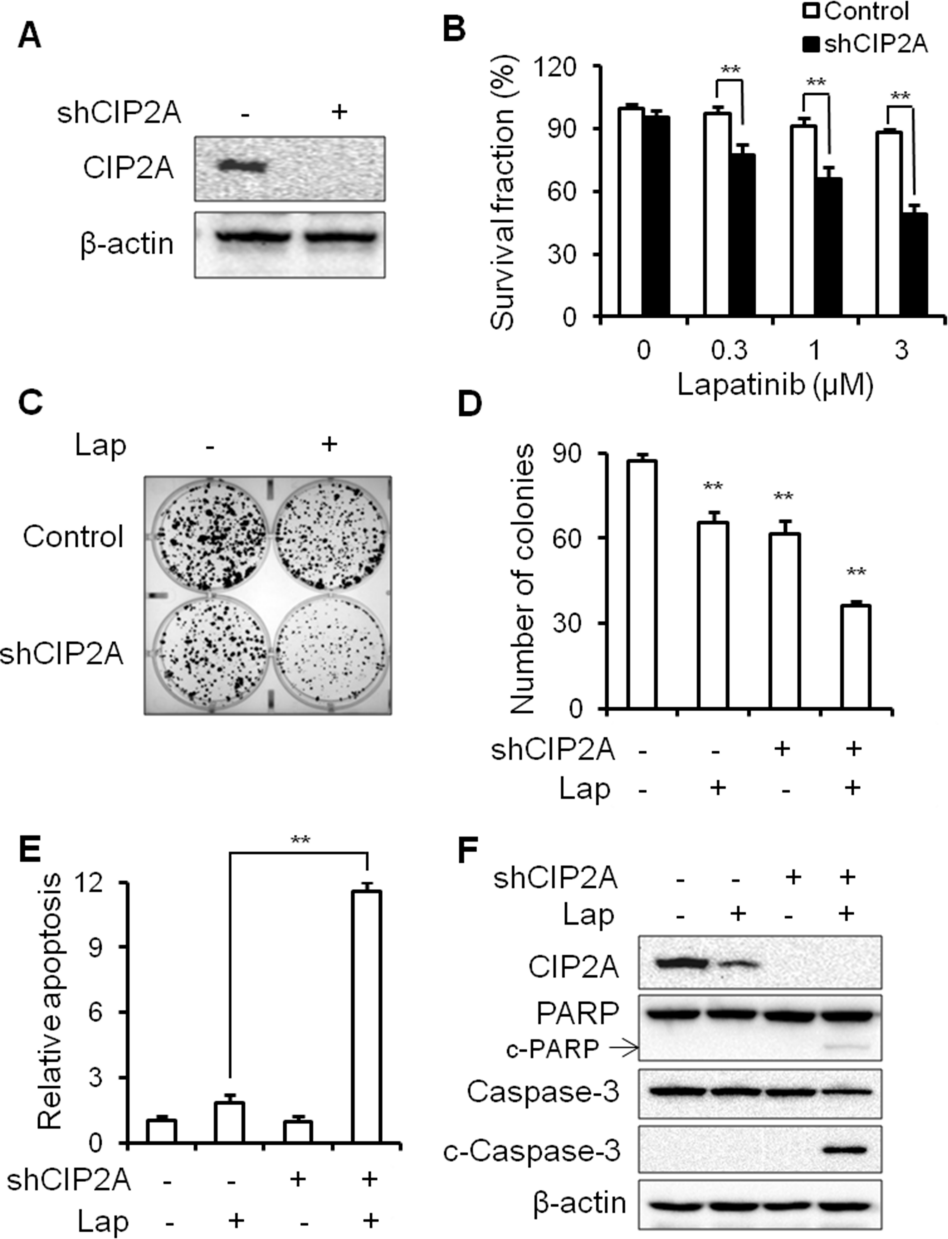
CIP2A knockdown can overcome lapatinib resistance in BT474/LR cells (**A**) Relative CIP2A levels in stable BT474/LR sublines transfected with control (–) or CIP2A shRNA-encoding (+) lentiviral vectors were analyzed with Western blotting. (**B**) Survival fractions in control and CIP2A knockdown BT474/LR cells treated with lapatinib for 5 days were measured with an MTS assay. Control and CIP2A knockdown BT474/LR cells were treated with lapatinib (0.03 μM) for 2 weeks and then clonogenic survival was determined by staining colonies with crystal violet. Representative images are shown in (**C**). The bar graph represents the mean values from three independent experiments (**D**). Control and CIP2A knockdown cells were treated with lapatinib (3 μM) for 48 hours and apoptosis was detected with a cell death ELISA assay (**E**) and Western blot analysis of PARP and Caspase-3 cleavage (**F**). All values are presented as the mean ± S.E. (**p < 0.01).

**Figure 9:**
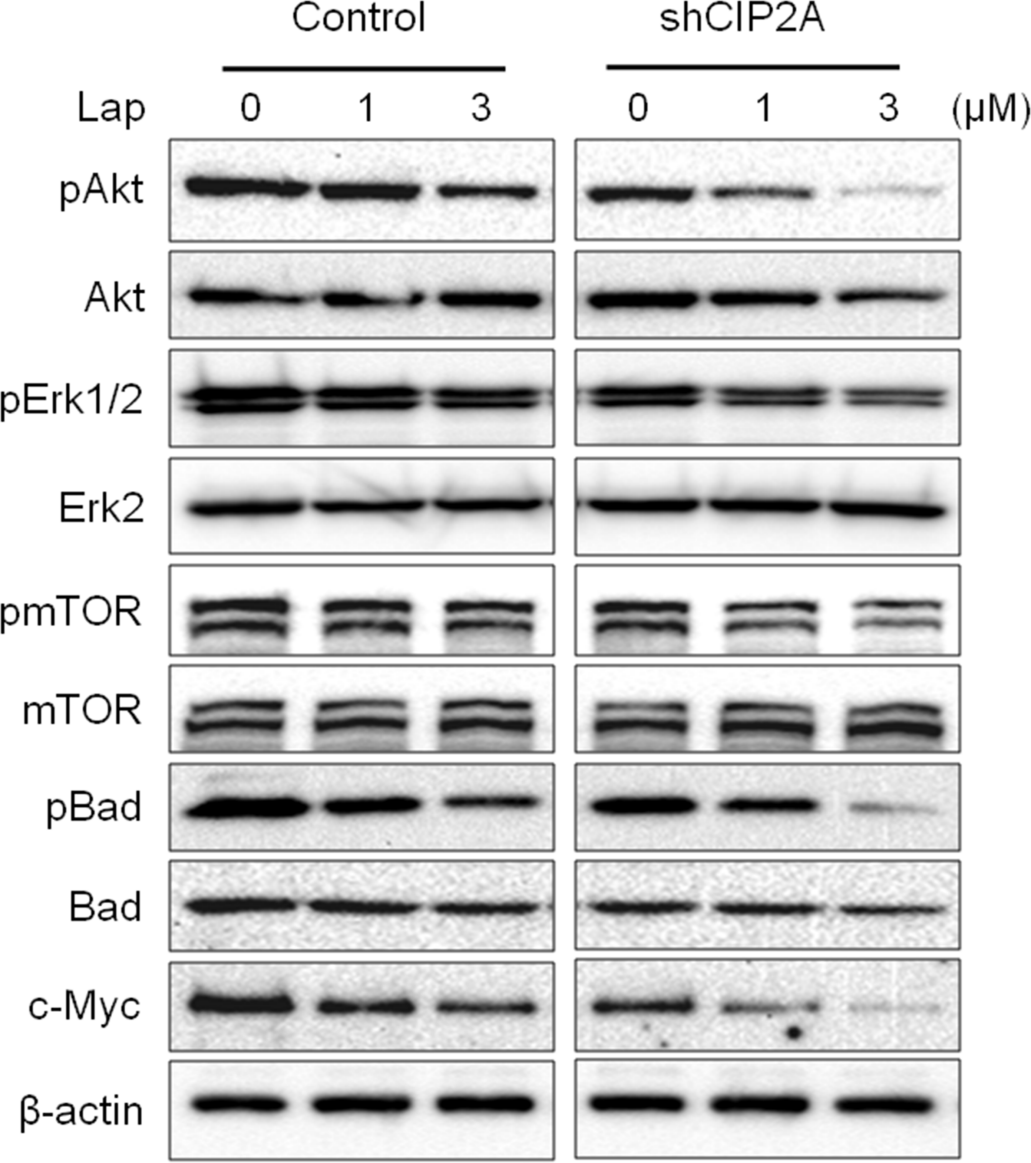
CIP2A knockdown sensitizes BT474/LR cells to lapatinib-induced inhibition of oncogenic signal transduction Control and CIP2A knockdown BT474/LR cells were treated with lapatinib for 16 hours and then analyzed for protein expression using Western blotting.

To confirm the specific role of CIP2A in lapatinib resistance, we examined the effect of CIP2A shRNA knockdown on lapatinib responsiveness in BT474/LR cells (Figure 8A). Indeed, CIP2A knockdown significantly sensitized BT474/LR cells to lapatinib, as reflected in both MTS and clonogenic assays (Figure 8B–8D). In the clonogenic experiments, CIP2A knockdown alone inhibited clone formation, while the combination of CIP2A knockdown and lapatinib treatment resulted in enhanced inhibition of the clonogenic formation capability in BT474/LR cells (Figure 8C–8D). In contrast, the BT474 parental cell line displayed a much stronger response to lapatinib- and/or shCIP2A-mediated clonogenic inhibition, as expected (Supplementary Figure 4). Moreover, we demonstrated that CIP2A knockdown in BT474/LR cells also sensitized the cells to lapatinib-induced apoptosis, as measured with a cell death ELISA assay (Figure 8E). Lapatinib-induced PARP and Caspase-3 cleavage was also synergistically enhanced in BT474/LR cells with CIP2A knockdown (Figure 8F). Due to the interaction of CIP2A with multiple pathways downstream of lapatinib-induced cellular responses, we examined the effect of CIP2A knockdown on lapatinib-induced regulation of Akt, Erk, mTOR, Bad, and c-Myc. As shown in Figure 9, lapatinib only triggered modest changes of the indicated markers in BT474/LR cells; however, CIP2A knockdown significantly diminished the expression of pAkt, pBad, and c-Myc in response to lapatinib treatment in BT474/LR cells. Some effects, although less evident, were also seen in pErk1/2 and pmTOR expression in response to lapatinib. Given the pivotal role of Akt, Bad, and c-Myc in the regulation of cell proliferation and survival, the differential responses between these two cell lines may explain CIP2A knockdown-mediated sensitization of BT474/LR cells to lapatinib. These results underscore the role of CIP2A deregulation in the development of lapatinib resistance and suggest that targeting CIP2A may be an effective strategy to overcome lapatinib resistance.

## DISCUSSION

In the present study, we found that lapatinib induces CIP2A downregulation in a concentration-dependent manner, which is also correlated with increased growth inhibition and apoptosis in the lapatinib-treated cells (Figure 1). As such, CIP2A overexpression significantly attenuated lapatinib-induced activities (Figure 2). Conversely, CIP2A knockdown through CIP2A-targeting shRNA enhanced cell sensitivity to lapatinib-induced growth inhibition and cell death (Figure 3), thus further demonstrating CIP2A as a critical intracellular target of lapatinib that mediates various cellular responses. These results advance our understanding of the mechanisms underlying lapatinib-induced anti-tumor activities. Although it is known that the immediate target of lapatinib is the ATP-binding pocket of ErbB2 and EGFR kinase domains, it has been demonstrated that modulation of specific intracellular targets can enhance or block lapatinib-induced anti-tumor activities. For example, lapatinib was reported to markedly inhibit the expression of survivin, an inhibitor of apoptosis protein (IAP), and enhance apoptosis in ErbB2-overexpressing breast cancer cells [34, 35]. It has also been shown that lapatinib-induced inhibition of the crosstalk between EGFR/ErbB2 and ER pathways in ErbB2^+^/ER^+^ breast cancers is closely linked to its anti-tumor efficacy. Our findings implicating CIP2A as a critical intracellular target of lapatinib are of similar significance. Importantly, we demonstrated that CIP2A upregulation is correlated with lapatinib resistance, which is of translational value for clinical oncology.

CIP2A was identified as an endogenous inhibitor of PP2A; therefore, our results demonstrating CIP2A as an intracellular target of lapatinib underscore the connection between lapatinib-mediated responses and the PP2A regulatory network. PP2A is a known tumor suppressor that inhibits many signaling pathways crucial for cell transformation. PP2A inactivation has been reported as a recurrent alteration in many types of cancer [22, 24–26]. In fact, suppression of PP2A appears to be associated with ErbB2-mediated carcinogenesis [26]. It was shown that inhibition of the PP2A catalytic subunit induced apoptosis through p38 MAPK, Caspase 3, and PARP activation in ErbB2-overexpressing breast cancer cells [36]. Given the broad-reaching impact of PP2A on cellular activities, such as maintenance of c-Myc stability, the connection between CIP2A and responsiveness to lapatinib may lead to the development of more strategies for the improvement of ErbB2/EGFR-targeting therapies. Identification of CIP2A as an intracellular target of lapatinib also supports CIP2A as a novel target in cancer therapy, as it is implicated in celastrol, erlotinib, and afatinib-induced anti-cancer effects [37–39]. Future combinational therapies using lapatinib and CIP2A-targeting agents may be promising breast cancer treatment strategies.

Our results indicated that lapatinib induces CIP2A downregulation at both the transcriptional and translational level, which is consistent with previous reports [30]. In our study, the mechanism by which lapatinib suppresses CIP2A mRNA expression is unclear; however, previous reports indicate that lapatinib can inhibit the binding of transcriptional activators, such as *Elk1*, to the *CIP2A* promoter [30]. While the mechanism of lapatinib-induced decrease in *CIP2A* mRNA levels requires further investigation, we focused on the post-translational regulation of CIP2A. As such, our data showed that lapatinib induced CIP2A degradation through the proteasomal pathway, as indicated by MG132-induced attenuation of CIP2A downregulation in lapatinib-treated cells (Figure 4). Since lapatinib-induced CIP2A downregulation was associated with concurrent inactivation of Akt, Erk, and mTOR (Figure 1), we used specific inhibitors targeting those pathways to test which inhibitor(s) mimic lapatinib-induced CIP2A downregulation. Among the three inhibitors, only Akt specific inhibitor LY294002 was able to downregulate CIP2A, similar to lapatinib treatment. Although mTOR activation is correlated with CIP2A deregulation [40, 41], mTOR inhibition via rapamycin treatment did not suppress CIP2A expression, indicating that CIP2A-induced mTOR activation observed in our study is likely a result of CIP2A-Akt interactions (Figure 4). Together, our results suggest that lapatinib-induced inactivation of the PI3K/Akt pathway stimulates consequential CIP2A downregulation, and Akt activity is a critical regulator of CIP2A. This is consistent with a report showing that celastrol, a natural compound with anti-cancer properties, significantly inhibits pAkt and targets CIP2A protein binding to the E3 ligase CHIP, which results in proteasomal degradation [38]. Other E3 ligases that have been found to target PP2A, such as DCAF1, Cullin3, and MID1 [42–44], may also be involved in proteasomal degradation of CIP2A due to the interaction between PP2A and CIP2A. Previously, it was also shown that CIP2A overexpression negatively regulates Akt-related PP2A activity and upregulates pAkt in hepatocellular carcinoma cells [29]. In our study, CIP2A knockdown reduced Akt phosphorylation in BT474/LR cells (Figure 9). Our data, in conjunction with previous reports, suggest that Akt and CIP2A form a positive feedback loop, and the interaction between Akt and CIP2A stimulates each other's protein levels and activities. Simultaneous downregulation of Akt and CIP2A in lapatinib-treated cells indicates that lapatinib induces the breakdown of the CIP2A-Akt feedback loop. Given the functional context of Akt and CIP2A in cellular regulation, lapatinib-induced interruption of the CIP2A-Akt feedback loop would lead to an extensive impact on its tumor inhibitory effects.

As suggested by the critical role of CIP2A in lapatinib-induced inhibition of ErbB2-overexpressing breast cancer cells, we further demonstrated that CIP2A deregulation was associated with lapatinib resistance. In contrast to the sensitive induction of CIP2A degradation in lapatinib-treated BT474 and 78617 cells, lapatinib failed to downregulate CIP2A in BT474/LR and 78617/LR cells, established cell line models of acquired lapatinib resistance (Figures 6, 7). It is important to note that lapatinib resistance in the BT474/LR and 78617/LR cells is relative to the parental cell lines; therefore, total lapatinib resistance is not necessary to observe the associated LR phenotypes. Based on our data from Figure 4 indicating the presence of a CIP2A-Akt feedback loop, CIP2A overexpression promotes Akt activation, which in turn can override lapatinib-mediated EGFR/ErbB2 signaling inhibition in lapatinib-resistant cells (Supplementary Figure 5). Importantly, CIP2A knockdown significantly sensitized the cells to lapatinib-induced growth inhibition and apoptosis (Figure 8). Enhanced inhibition of Akt, Bad, and c-Myc phosphorylation/activation in lapatinib-treated BT474/LR cells with CIP2A knockdown provided further support for the intrinsic connection between CIP2A modulation and lapatinib sensitivity (Figure 9). These findings underscore CIP2A as a candidate risk factor contributing to lapatinib resistance.

Given the clinical use of lapatinib as a major therapeutic agent for ErbB2^+^ breast cancers, lapatinib resistance has emerged as a significant clinical challenge. To this end, our current study investigates potential factors that may contribute to lapatinib resistance. In this regard, multiple mechanisms of lapatinib resistance have previously been studied in preclinical and clinical models, including heregulin-driven ErbB3/EGFR-PI3K signaling axis, altered expression of pro- and anti-apoptotic proteins (e.g. survivin), and activation of β1 integrin or AXL [18, 35, 45]. Our identification of CIP2A as a novel intracellular target and resistance factor has a multi-fold impact on lapatinib therapy and resistance. First, we demonstrated the presence of a feedback loop between CIP2A and Akt in lapatinib-induced cellular responses. In addition to the common factors that cause deregulation of the RTK-PI3K-Akt axis, such as PTEN and Akt mutations, our results added a new mechanism that contributes to Akt hyperactivation and drug resistance. Second, CIP2A is an intrinsic inhibitor of PP2A; therefore, the connection between CIP2A and lapatinib sensitivity highlights the role of the crosstalk between PI3K/Akt pathway and the PP2A network in carcinogenesis and drug response. It also suggests that exploring whether deregulation of other PP2A inhibitors, such as SET [46], in lapatinib-mediated responses is warranted. Third, given the prevalent incidence of upregulated CIP2A and Akt in various cancers [24, 47], our understanding of CIP2A and lapatinib resistance may also shed light on the mechanisms of the resistance to other ErbB2-targeting drugs, such as trastuzumab. In particular, CIP2A overexpression has been reported in approximately 60% of ErbB2^+^ breast cancer samples and may have potential as a clinical predictor for lapatinib responsiveness [48]. Fourth, our study also suggests that combining CIP2A-targeted approaches with lapatinib therapy would be a useful strategy to overcome lapatinib resistance, although current small molecules targeting CIP2A have not been developed.

In summary, we showed that CIP2A is a critical intracellular target of lapatinib. Lapatinib induces growth inhibition and apoptosis in ErbB2-overexpressing breast cancer cells by breaking the CIP2A-Akt feedback loop. We demonstrated that upregulation of CIP2A contributes to lapatinib resistance, and the specific targeting of CIP2A sensitizes lapatinib-resistant cells to lapatinib. Our study highlights the connection between the ErbB2-Akt and CIP2A-PP2A regulatory networks. The data provide evidence for CIP2A as a risk factor for acquired lapatinib resistance, thus making it a potential target for novel breast cancer therapies.

## MATERIALS AND METHODS

### Reagents

Lapatinib was purchased from LC Laboratories (Woburn, MA). MG132, CHX, RNase A, and propidium iodide (PI) were purchased from Sigma-Aldrich (St. Louis, MO). LY294002 and PD98059 were purchased from Alexis Biochemicals (San Diego, CA). EGF was purchased from Peprotech (Rocky Hill, NJ). Rapamycin and antibodies against pErbB2 (Tyr1221/1222), ErbB2, pEGFR (Tyr1068), EGFR, pmTOR (Ser2448), mTOR, pAkt (Ser473), Akt, pBad, Bad, and PARP were purchased from Cell Signaling Technology (Danvers, MA). Antibodies against CIP2A, c-Myc, pErk1/2 (Thr202/Tyr204), Erk2, Caspase-3, cleaved Caspase-3, cyclin D1, and β-actin were purchased from Santa Cruz Biotechnology (Santa Cruz, CA).

### Cell culture

SKBR3 and BT474 cell lines were purchased from the American Type Culture Collection (ATCC; Manassas, VA). The 78617 cell line was derived from mammary tumor cells developed in MMTV-ErbB2 transgenic mice, as described in our previous reports [31]. The lapatinib-resistant sublines, BT474/LR and 78617/LR, were generated by continuous exposure of parental BT474 and 78617 cells to gradually increasing concentrations of lapatinib. The entire process of resistance training took over 5 months to obtain the BT474/LR and 78617/LR cell lines used in this study. Briefly, the process of resistance training involved the treatment of BT474 and 78617 cells with increasing doses of lapatinib (0.1, 0.5, 1, 2, and 4 μM). Cells exhibiting lapatinib resistance were allowed to stably proliferate before each incremental dose increase. All cells were cultured in DMEM/F12 medium supplemented with 10% fetal bovine serum, 100 units/mL penicillin, and 100 μg/mL streptomycin and incubated in a 5% CO_2_ atmosphere at 37°C.

### Cell viability (MTS) assay

Cell viability was measured using the CellTiter 96 AQueous Non-Radioactive Cell Proliferation kit (Promega; Madison, WI). Briefly, cells were seeded in 96-well plates at a density of 2000 cells/well in 100 μL of complete medium. The following day, the cells were treated with indicated concentrations of lapatinib for 5 days. At the end point, 20 μL of the MTS/PMS solution was added to each well. After incubation for 2 hours, the absorbance was quantified at 490 nm with a SynergyMx microplate reader (BioTek; Winooski, VT). Cell viability was expressed as the percentage of viable cells treated with lapatinib as compared to the DMSO-treated control. All experiments were performed in triplicate.

### Cell cycle assay

Cells were harvested and washed twice with PBS, and then fixed in 70% ethanol at -20°C overnight. The cells were then washed with PBS and incubated with RNase A (0.5 mg/mL) and PI (50 μg/mL) at 37°C for 45 minutes. The cells were analyzed with a Guava easyCyte 8 flow cytometer (Millipore), and the percentage of cells at each phase of the cell cycle was determined using ModFit software.

### Apoptosis ELISA

Apoptosis of the treated cells was detected with a Cell Death Detection ELISA kit (Roche Life Science; Indianapolis, IN). The treated cells were collected and counted with a hemocytometer. Cells (1 × 10^4^/sample) were lysed in 200 μL buffer for 30 minutes, followed by centrifugation. An aliquot of supernatant (20 μL) was transferred into a microplate to react with the immunoreagents. The wells were washed and developed before the absorbance was measured at 405 nm with a SynergyMx microplate reader. The relative apoptosis in the treated groups was expressed as a ratio as compared to the control group. The experiments were performed in triplicate.

### Clonogenic assay

Single cell suspensions were prepared by trypsinization, followed by cell counting with a hemocytometer. The cells were seeded into 6-well plates at 500–1000 cells/well and treated with indicated doses of lapatinib for two weeks. The plates were then fixed with methanol and stained with 0.1% crystal violet. The images were captured using a FluorChemE system (Cell Biosciences; Santa Clara, CA).

### Lentiviral production and infection

The CIP2A-encoding lentiviral vector pReceiver-Lv105 was purchased from GeneCopoeia (Rockville, MD). The lentiviral envelope vector pMD2.G and packaging vector psPAX2 were ordered from Addgene (Cambridge, MA). For lentivirus production, CIP2A- or GFP-encoding lentiviral vectors and pMD2.G/psPAX2 plasmids were co-transfected to 293T cells using X-tremeGENE 9 transfection reagent (Roche Diagnostics) according to the manufacturer's instructions. The stocks of GFP/control and CIP2A-expressing lentiviruses were collected at 24 and 48 hours after transfection and concentrated by ultracentrifugation. The viral titer was determined by infection of 293T cells with serial dilutions of the viral stock. For CIP2A knockdown experiments, copGFP control shRNA and CIP2A shRNA lentivirus particles were purchased from Santa Cruz Biotechnology. For viral infection, cells were seeded in 60 mm dishes at 1 × 10^6^ cells per dish 24 hours prior to infection, followed by infection of specific viruses at 10 MOI/cell in the presence of polybrene (5 μg/mL). For transient transfection experiments, cells were treated with lapatinib after 48 hours of infection. For stable cell lines, the infection efficiency was detected by monitoring GFP expression after 24 hours of infection.

### Real-time (RT)-PCR

RNA was extracted with TRIzol reagent (Life Technologies; Carlsbad, CA) following standard RNA extraction protocol. One μg of total RNA was reverse transcribed using MMLV reverse transcriptase (Bio-Rad; Hercules, CA) and the resulting cDNA was used for qRT-PCR reactions with SYBR Green qPCR Mastermix (Qiagen; Valencia, CA). The samples were amplified in 25 μL reactions with gene specific primers. The primers used for amplification were as follows: *GAPDH* Forward: 3′-TGC ACC ACC AAC TGC TTA GC-5′, Reverse: 3′-GGC ATG GAC TGT GGT CAT GAG-5′; and *CIP2A* Forward: 3′-GAA CAG ATA AGA AAA GAG TTG AGC ATT-5′, Reverse: 3′-CGA CCT TCT AAT TGT GCC TTT T-5′. Each reaction mixture was amplified in triplicate and the results calculated based on the ΔΔCt method. The cycle threshold (Ct) value for *CIP2A* gene expression was normalized using the mean Ct value for *GAPDH* gene expression. Relative gene expression was expressed as the fold change (2^–ΔΔCt^) relative to expression in the untreated control.

### Western blotting

The collected cells were lysed in Laemmli sample buffer (Bio-Rad). Whole cell lysates were boiled for 10 minutes and then centrifuged for 10 minutes to remove cellular debris. The protein concentrations were quantified with a Pierce BCA Protein Assay kit (Thermo Scientific; Rockford, IL). Fifty μg of total protein were separated on 8%–12% sodium dodecyl sulfate-polyacrylamide gel electrophoresis (SDS-PAGE) and transferred onto polyvinyl difluoride (PVDF) membranes. The membranes were blocked at room temperature for 1 hour in 5% nonfat dry milk, incubated with the indicated primary antibodies overnight at 4°C, washed in TBST buffer, and then incubated for 1 hour with appropriate horseradish peroxidase (HRP)-conjugated secondary antibodies at room temperature. The membranes were washed in TBST buffer again and immune complexes were finally detected with SuperSignal West Pico stable peroxide solution (Thermo Scientific). The protein bands were visualized using FluorChemE imager.

### Syngeneic tumor cell transplantation

Female MMTV-ErbB2 transgenic mice were purchased from Jackson Laboratories (Bar Harbor, ME). All mice were fed a standard, estrogen-free AIN-93G diet (Bio-Serv; Flemington, NJ). At 8 weeks of age mice were subcutaneously inoculated with 78617 or 78617/LR cells (5 × 10^5^) in each flank. Then, when tumors were palpable, vehicle or lapatinib treatments (75 mg/kg BW twice daily) were administered via oral gavage beginning at Day 6 following the tumor cell inoculations. Tumors were monitored every 2 days by palpation beginning at 6 days after the tumor cell inoculations and tumor volumes [(width^2^ × length)/2] were recorded. After 4 days of lapatinib treatments, tumors were collected and analyzed for CIP2A protein expression and Akt phosphorylation in a cohort of vehicle- and lapatinib-treated mice. On Day 20 after the initial tumor cell inoculations, the remaining tumors were excised and weighed. All animal procedures were approved by the Institutional Animal Care and Use Committee.

### Statistical analysis

The results are presented as the means ± standard error (S.E.) of at least three separate experiments. The differences between groups were evaluated by Student's *t*-test or one-way analysis of variance (ANOVA) followed by Dunnett's test for multiple comparisons. Analyses were done with GraphPad Prism software (La Jolla, CA). The levels of significance were defined as: *p < 0.05, **p < 0.01.

## SUPPLEMENTARY MATERIALS METHODS AND FIGURES



## Abbreviations

ANOVA: Analysis of variance
CIP2A: cancerous inhibitor of protein phosphatase 2A
ct: cycle threshold
CHX: cycloheximide
EGFR: epidermal growth factor receptor
ErbB2^+^: ErbB2-positive
ER: estrogen receptor
HRP: horseradish peroxidase
IAP: inhibitor of apoptosis protein
LR: lapatinib-resistant
PVDF: polyvinyl difluoride
PI: propidium iodide
PP2A: protein phosphatase 2A
RT-PCR: real-time polymerase chain reaction
RTK: receptor tyrosine kinase
SDS-PAGE: sodium dodecyl sulfate-polyacrylamide gel electrophoresis
S.E.: standard error
