# Supplementary material for: Activation of cancerous inhibitor of PP2A (CIP2A) contributes to lapatinib resistance through induction of CIP2A-Akt feedback loop in ErbB2-positive breast cancer cells
Source: Oncotarget. 2017 Jul 19;8(35):58847–64. doi: 10.18632/oncotarget.19375 (PMC5601698; doi:10.18632/oncotarget.19375)
Supplement: Supplementary file 1 [file oncotarget-08-58847-s001.pdf]

Activation of cancerous inhibitor of PP2A (CIP2A) contributes to lapatinib resistance through induction of CIP2A-Akt feedback loop in ErbB2-positive breast cancer cells

SUPPLEMENTARY MATERIALS

MATERIALS AND METHODS

Densiometry

The optical densities (OD) of detected protein bands from 3 independent experiments were quantified using Quantity One software (Bio-Rad). The relative band densities were calculated as the OD ratio of the detected proteins relative to the β-actin loading control from the same sample. The control sample OD ratios were normalized to 1.

Immunoprecipitation

After MG132 and/or lapatinib treatments, cells were washed twice with ice-cold PBS. Then, cells were incubated on ice for 30 minutes in lysis buffer supplemented with a protease inhibitor cocktail (Sigma). Following measurement of protein concentration, total protein extracts (500 μg) were incubated with the anti-CIP2A primary antibody overnight at 4°C. Next, samples were incubated in Protein G PLUS-Agarose (Santa Cruz) for 2 hours at 4°C while rotating. The beads were then washed five times with 1 ml lysis buffer and the immunoprecipitated proteins were isolated by boiling in sample buffer. After protein separation with SDS-PAGE, ubiquitinated CIP2A protein levels were detected by standard Western blotting techniques using an anti-ubiquitin primary antibody (Cell Signaling Technology). CIP2A protein levels serve as an internal loading control.

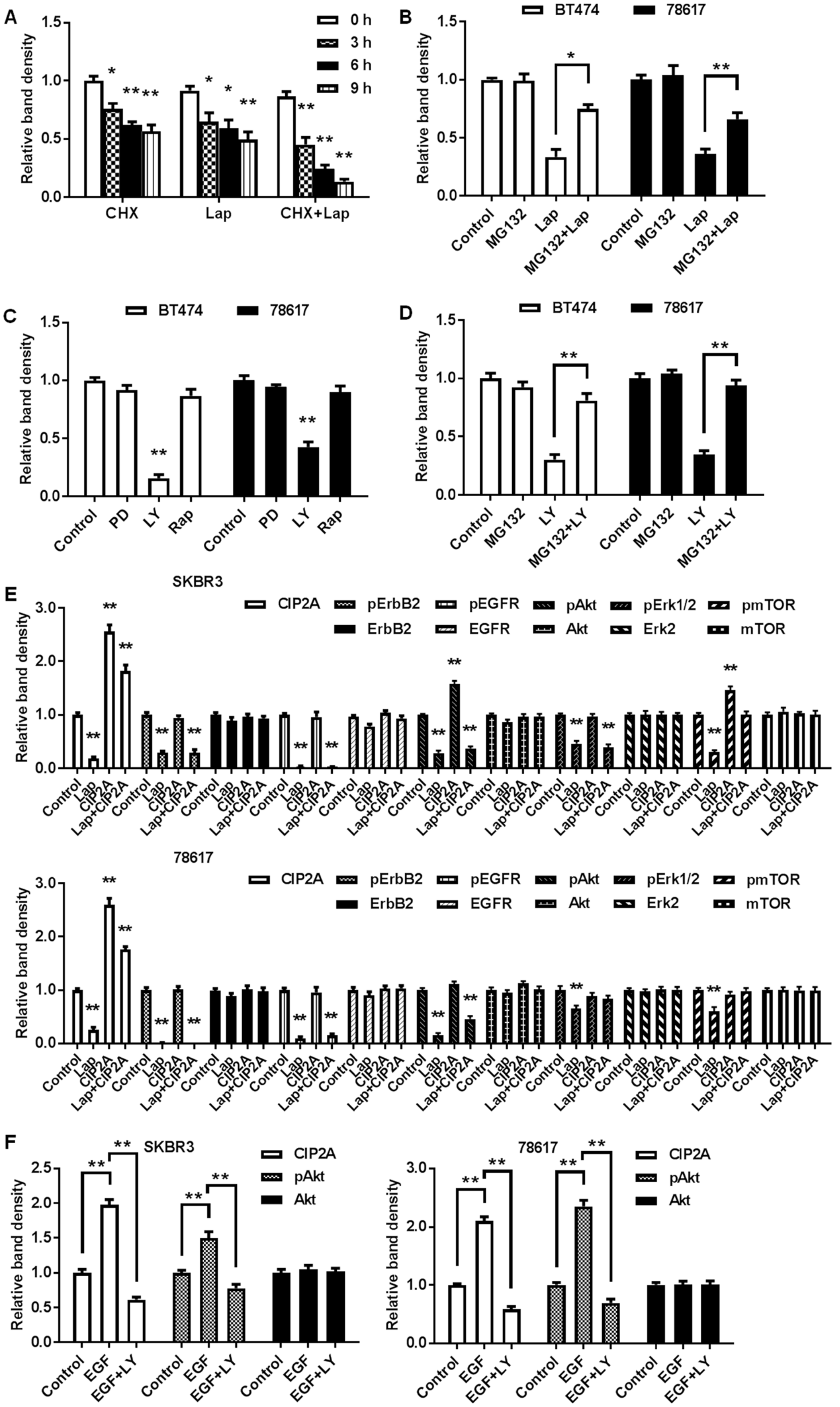

Supplementary Figure 1: Western blot quantification. (**A–F**) Western blots from Figure 4B–4G were quantified based on the optical densities of detected bands from at least 3 independent experiments. All values are graphed as the mean ± S.E (*$p < 0.05$, **$p < 0.01$ as compared to the corresponding control unless indicated otherwise).

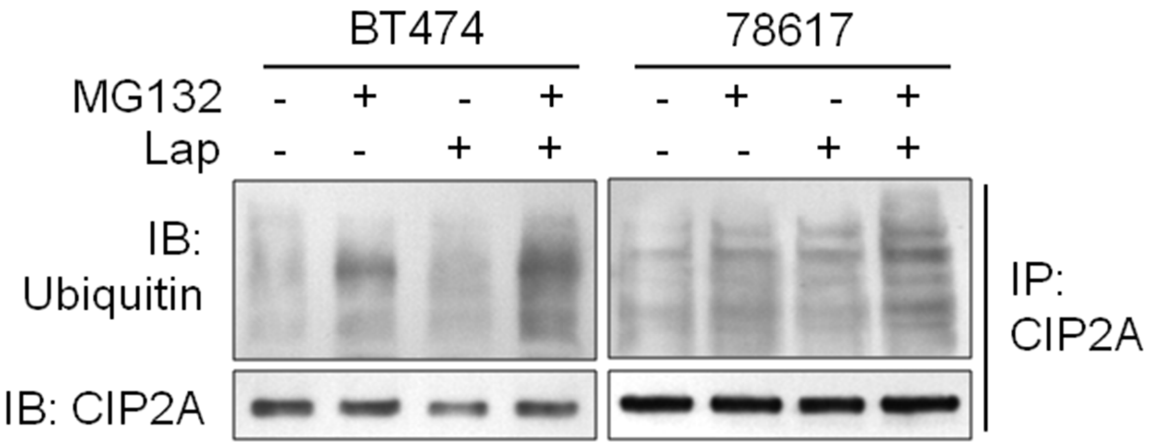

Supplementary Figure 2: Lapatinib degrades CIP2A through the proteasomal pathway. BT474 and 78617 cells were pretreated with MG132 (2 μM) alone for 1 hour, lapatinib (0.3 μM) alone for 12 hours, or in combination, then CIP2A was immunoprecipitated, followed by immunoblotting of ubiquitinated proteins.

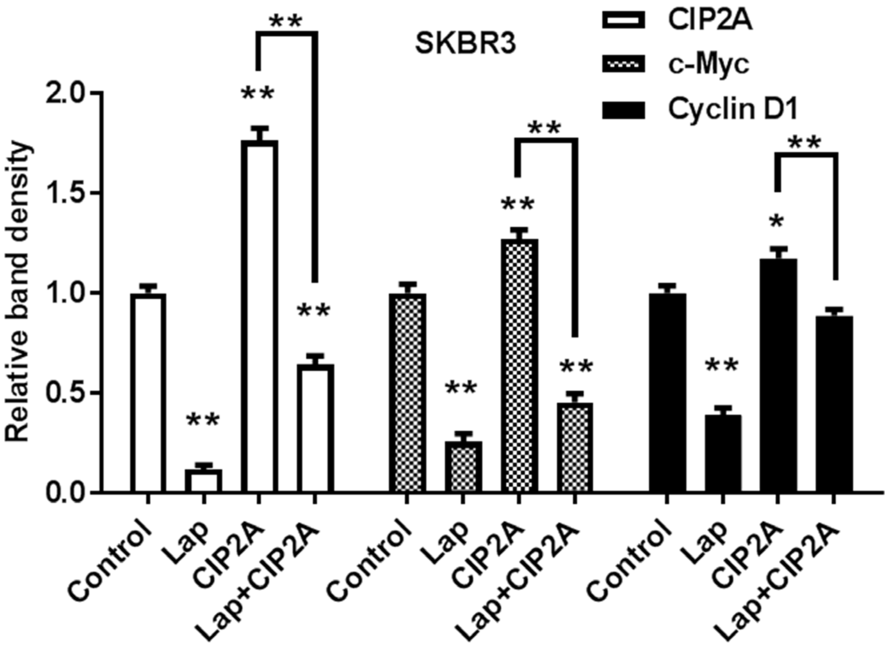

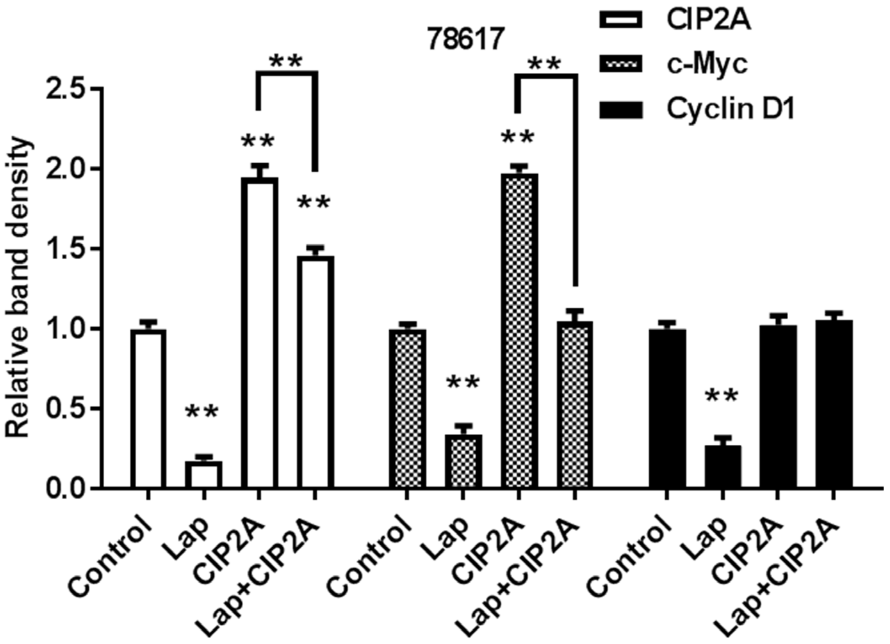

Supplementary Figure 3: CIP2A overexpression induces c-Myc and cyclin D1. Western blots from Figure 5B were quantified based on the optical densities of detected bands from at least 3 independent experiments. All values are graphed as the mean ± S.E (*$p < 0.05$, **$p < 0.01$ as compared to the corresponding control unless indicated otherwise).

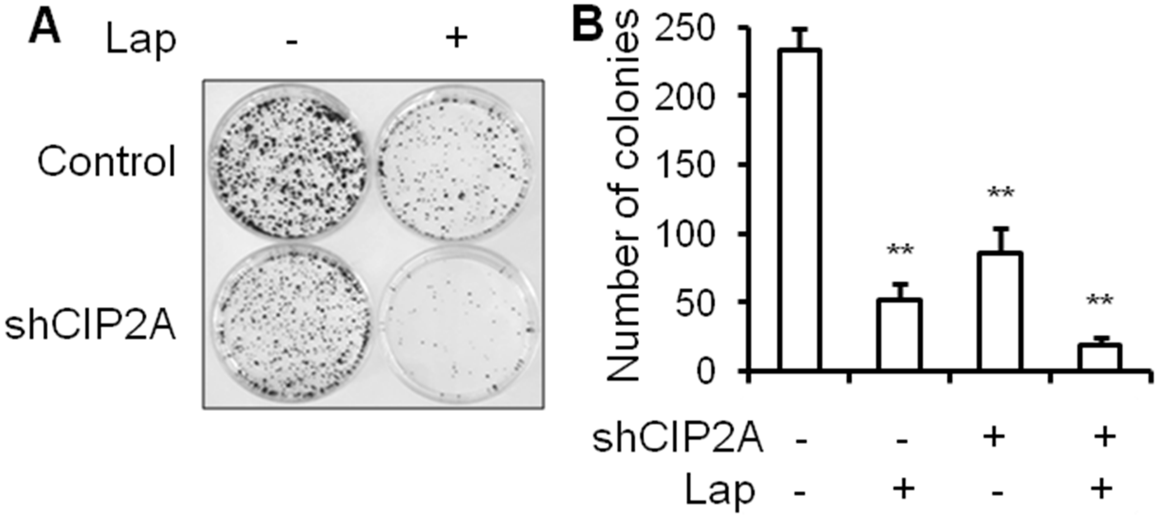

Supplementary Figure 4: CIP2A knockdown suppresses colony formation in BT474 parental cells. Control and CIP2A knockdown BT474 cells were treated with lapatinib (0.03 μM) for 2 weeks and then clonogenic survival was determined by staining colonies with crystal violet. Representative results are shown in (**A**). The bar graph represents the mean values from three independent experiments (**B**). Values are presented as the mean ± S.E. (**$p < 0.01$).

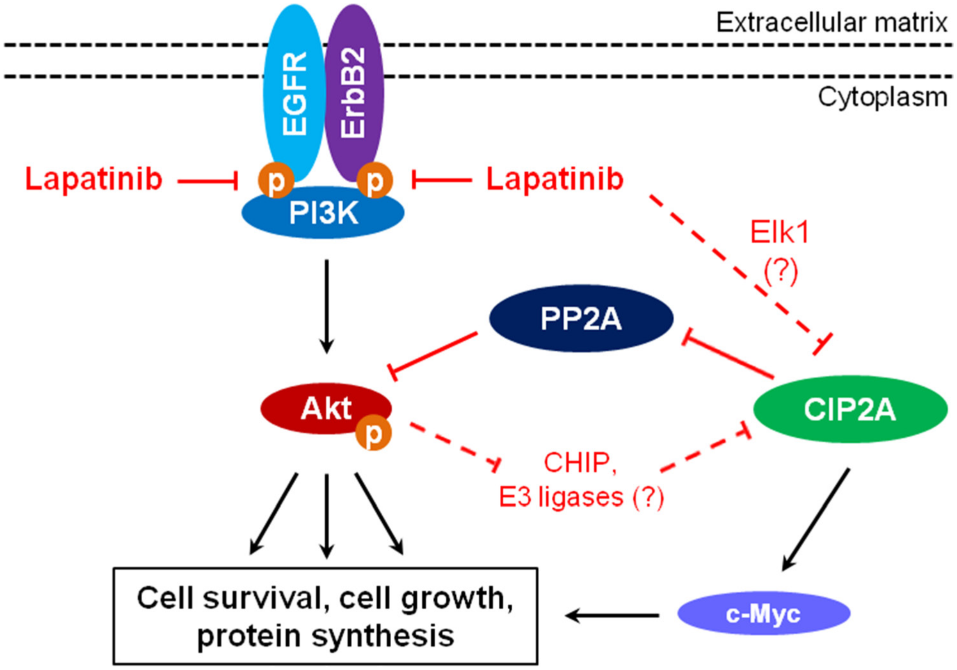

Supplementary Figure 5: Proposed model of CIP2A-Akt feedback loop inhibition in lapatinib-responsive cells. Lapatinib blocks the CIP2A-Akt feedback loop through the inhibition of EGFR/ErbB2 receptor activation and subsequent downstream signaling. Also, lapatinib can indirectly inhibit CIP2A via Elk1 or other unknown mechanisms.
